# Molecular and Genetic Determinants of Glioma Cell Invasion

**DOI:** 10.3390/ijms18122609

**Published:** 2017-12-04

**Authors:** Kenta Masui, Yoichiro Kato, Tatsuo Sawada, Paul S. Mischel, Noriyuki Shibata

**Affiliations:** 1 Department of Pathology, Tokyo Women’s Medical University, Tokyo 162-8666, Japan; katoyo@twmu.ac.jp (Y.K.); sawada.tatsuo@twmu.ac.jp (T.S.); shibatan@twmu.ac.jp (N.S.); 2 Ludwig Institute for Cancer Research, University of California San Diego, La Jolla, CA 92093, USA; pmischel@ucsd.edu

**Keywords:** glioma cell invasion, microenvironment, isocitrate dehydrogenase (IDH), mammalian target of rapamycin complex 2 (mTORC2), metabolic reprogramming

## Abstract

A diffusely invasive nature is a major obstacle in treating a malignant brain tumor, “diffuse glioma”, which prevents neurooncologists from surgically removing the tumor cells even in combination with chemotherapy and radiation. Recently updated classification of diffuse gliomas based on distinct genetic and epigenetic features has culminated in a multilayered diagnostic approach to combine histologic phenotypes and molecular genotypes in an integrated diagnosis. However, it is still a work in progress to decipher how the genetic aberrations contribute to the aggressive nature of gliomas including their highly invasive capacity. Here we depict a set of recent discoveries involving molecular genetic determinants of the infiltrating nature of glioma cells, especially focusing on genetic mutations in receptor tyrosine kinase pathways and metabolic reprogramming downstream of common cancer mutations. The specific biology of glioma cell invasion provides an opportunity to explore the genotype-phenotype correlation in cancer and develop novel glioma-specific therapeutic strategies for this devastating disease.

## 1. Introduction—Invasion as a Key Feature in Gliomas

Recent advances in the identification of detailed genetic and epigenetic profiling in diffuse gliomas have led to the refinement of glioma classification [1,2]. However, current therapeutics for diffuse gliomas are still inadequate, and the patients eventually succumb to the disease despite the combination of treatment options for diffuse glioma. One of the main reasons for this therapeutic failure could be attributed to a key characteristic of glioma cells to vigorously infiltrate adjacent brain tissue, which is responsible for the term “diffuse” glioma. The highly invasive capacity of diffuse glioma cells prevents total resection of the tumor during surgery, and the investigation of the mechanism of glioma cell invasion has thus received a great deal of interest in the field.

From a histopathological standpoint, infiltration of glioma cells has been a well-recognized characteristic for the diagnostics of diffuse gliomas. For instance, glioma cells migrate along existing brain structures including the brain parenchyma (especially around neuronal cells), blood vessels, white matter tracts and subpial spaces, so-called “Scherer’s secondary structures”, reported by Hans Joachim Scherer, a German neuropathologist in 1938 [3]. Careful observations of these histological features of glioma invasiveness have revealed the important contribution of microenvironment in the tumor to support glioma cell migration. Further, the recent molecular biological approaches have unraveled the underlying mechanism of the infiltrating nature of glioma cells, which cleverly use the intracellular systems originally residing in the migratory neural constituents in the brain. It would be thus important that future endeavor be directed to examine the connection between genetic/epigenetic aberrations and biochemical functions including cellular invasion in glioma cells.

We herein review a set of recent discoveries involving the aggressive infiltrating nature of glioma cells. A systematic search of PubMed for the literature analyses was performed between 1999 and 2017 with a combination of the keywords: “glioma”, “glioblastoma”, “invasion”, “migration”, “molecular”, “genetic” and “metabolism”, and reference articles were also garnered through the authors’ own file collections. We especially focus on intracellular molecular machineries to drive migratory activity of glioma cells as well as their intricate interaction with the microenvironmental components. We also discuss how the infiltrating nature could be promoted by genetic mutations in receptor tyrosine kinase (*RTK*) pathways, which highlight the integration of genetic aberrations with altered signaling, metabolic reprogramming, and epigenetic changes downstream of common cancer mutations, potentially providing new therapeutic opportunities for these deadly types of brain tumors.

## 2. Molecular Underpinnings of Glioma Cell Invasion

### 2.1. Exploitation of Migratory Traits of Existent Neural Cells

**Figure 1:**
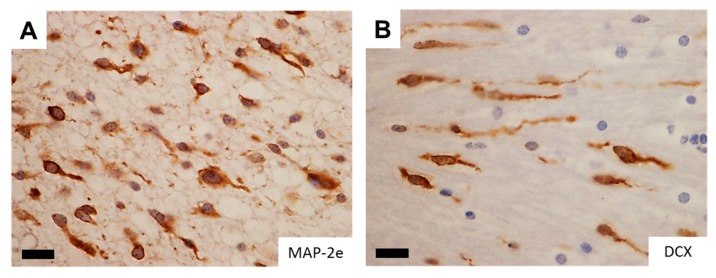

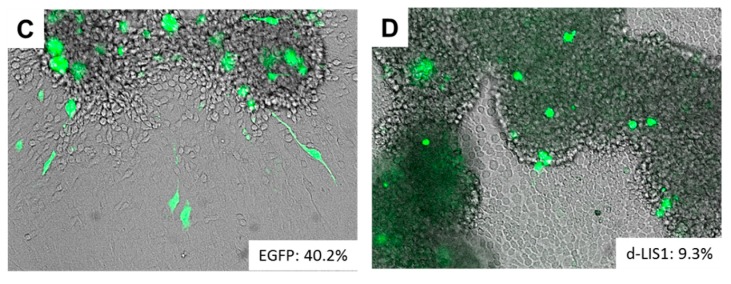
Neuro-developmental gene products in infiltrating glioma cells. (**A**,**B**) Microtubule-associated proteins, which are important in migration of glial ((**A**) MAP-2e) and neural ((**B**) DCX) progenitor cells clearly delineate infiltrating glioma cells, well demonstrating the leading processes. Images reproduced from [7]. Scale bar = 15 µm; (**C**,**D**) GFP-labeled C6 rat glioma cell lines vigorously migrate out of the sphere ((**C**) 40.2%) whereas overexpression of dominant negative form of neuro-developmental gene LIS1 (d-LIS1) significantly reduced the ratio of migration ((**D**) 9.3%), suggesting its role in glioma cell invasion. MAP-2e, microtubule-associated protein-2e; DCX, doublecortin; EGFP, enhanced green fluorescent protein; d-LIS1, dominant negative form of lissencephaly-1.

A significant analogy has traditionally been recognized between glioma cell invasion and the migration pattern of normal neural progenitor cells during development. To morphologically support this notion, studies with time-lapse imaging of neural progenitor cells and glioma cells migrating ex vivo in the brain slices demonstrated remarkable similarity in the morphology and dynamics of migration patterns where the cells protrude a leading process before the nuclear translocation [4,5,6]. The observation suggests a role of microtubules and associated proteins in the migration of active progenitor and glioma cells and raises their potentiality as markers for detecting infiltrating glioma cells in the brain. Of note, DCX (doublecortin) and LIS1 (lissencephaly-1), the causative genes for X-linked lissencephaly and type 1 lissencephaly respectively, are expressed in infiltrating glioma cells (Figure 1), delineating the infiltrating glioma cells in the brain comparable to the expression pattern of MAP-2e, a splicing variant of MAP-2 (microtubule-associated protein-2) that has been shown to detect glioma invasion into the adjacent brain tissue [7,8,9]. Disruption of these gene products significantly retards the migration of glioma cells (Figure 1). The findings are compatible with the assumption that these developmental genes could play a role in tumor cell invasion, analogous to their roles in neural progenitors during brain development [10].

Actin–myosin molecular motors provide the main contractile force in intrinsic neural cells, and thus contribute to the support of cell migration. Previous studies demonstrated that invasive glioma cells use non-muscle myosin II to generate the contractile forces to squeeze their cell bodies through the small intercellular spaces that characterize brain white matter and cortex, promoting the migration of glioma cells [11,12,13]. Cellular migration with this system is thus responsive to environmental stiffness within tumor tissue to achieve maximal migration capability [14,15] with an additional support of ion and water channels [3,16]. Additionally, microtubule-associated motor proteins including kinesin and dynein, which play essential roles in intracellular transport and in the formation of the mitotic spindle apparatus, are also reported to be involved in the infiltrative capacity of glioma cells and could be the therapeutic and diagnostic targets [9,17].

### 2.2. Interaction with Tumor Microenvironment

Tumor cells do not solely rely on their own migratory machineries, but on the interaction with surrounding microenvironment to support their invasive capacity. Various components exist within or around the tumor including blood vessels, neuropils consisting of dendrites and glial processes, and the white matter axonal tracts. Glioma cells display vigorous avidity for the extracellular matrix (ECM) constituting these microenvironmental components, and the close interaction with ECM components is inevitable for the efficient migration of glioma cells in the brain. As aforementioned, the protrusion of a leading process precedes the nuclear translocation in cellular migration, which is an integrated process with a cascade of projection of the leading edge from a migrating cell, anchoring to the ECM, and detachment of the trailing end [18]. Cell attachment is mediated by cell–cell and cell–matrix receptors, such as integrins, cadherins and cell adhesion molecules (CAMs) [19]. Integrins are transmembrane receptors involved in cell–cell and cell–matrix interactions, and glioma cells display several integrin family members with β1 integrin playing a central role in glioma invasion [20], by activating tyrosine kinases such as focal adhesion kinase (FAK) [21]. Cell detachment requires the activity of proteases that degrade ECM components, such as matrix metalloproteinases (MMPs). Glioma cells secrete an array of proteases to be involved in glioma cell invasion, including MMP2 and MMP9 [22,23], and the membrane-bound MMP, MT1-MMP (also known as MMP-14) [24]. The intricate interaction between glioma cells and ECM with the cellular receptors and proteases enables tumor cells to remodel the microenvironment to favor tumor cell invasion.

Microglia/macrophage is one of the immune cells in the brain that has been reported to constitute a significant subpopulation in the microenvironment of malignant tumors. These tumor-infiltrating microglia/macrophages are collectively referred to “tumor-associated macrophages” (TAMs). TAMs could be recruited in the tumor from resident brain microglia and monocyte-derived macrophages from the circulation, and glioblastoma (GBM), the most malignant astrocytic tumor, is often heavily infiltrated by such cells of myeloid origin [25]. TAMs have been implicated in several roles in GBM progression including proliferation, survival, immunosuppression and motility [26,27]. Various studies have sought to determine the role of TAMs in migration and invasion of glioma cells. It was reported that microglia can secrete a soluble motogenic factor that acts on glioma cells and substantially enhance the migration of tumor cells [28]. Further, microglia and glioma cells cooperate in the tumor tissue to secrete and activate MMPs, which mediate the breakdown of ECM required for invasion of the tumor [29].

## 3. Cardinal Regulators of Glioma Invasiveness—Genetic and Metabolic Aberrations Drive Glioma Cell Invasion

### 3.1. Invasive Phenotypes in IDH-Mutant Gliomas

**Figure 2:**
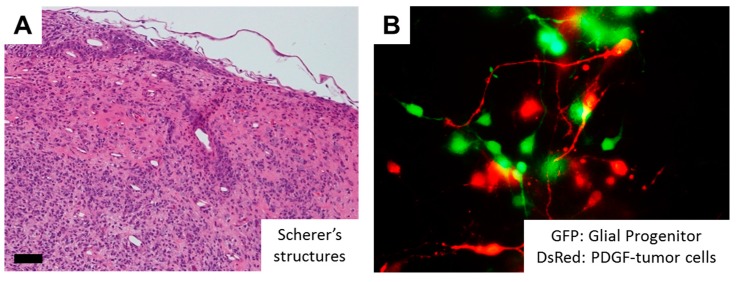

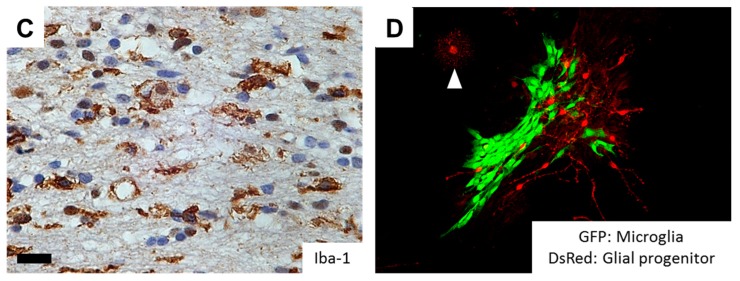
RTK signaling as a key determinant of glioma cell invasion. (**A**) In PDGF (platelet-derived growth factor)-induced rat glioma models, tumor cells demonstrate highly invasive features, so-called “Scherer’s secondary structures.” Scale bar = 50 µm; (**B**) Co-culture of PDGF-induced tumor cells (red) and NG2-positive glial progenitors (green) transforms glial progenitors into migratory morphology in a paracrine fashion; (**C**) In PDGF-induced rat glioma models, significant number of Iba-1-positive microglia are observed in the invasive front of the tumor. Scale bar = 15 µm; (**D**) Co-culture of microglial cell lines (green) and NG2-positive glial progenitors (red) from the rat brain transforms glial progenitors into migratory morphology, suggesting pro-invasive role of microglia. Note that the mature glial progenitor cells display oligodendroglial morphology with multipolar branches (arrowhead). GFP, green fluorescent protein; DsRed, Discosoma species red fluorescent protein; PDGF, platelet-derived growth factor; Iba-1, ionized calcium binding adapter molecule 1.

One of the epoch-making discoveries in glioma pathogenesis is the identification of hotspot mutations in isocitrate dehydrogenase 1 (*IDH1*), or less commonly *IDH2* genes in more than 70% of diffusely infiltrating World Health Organization (WHO) grade II and grade III astrocytic and oligodendroglial gliomas, as well as in a minor fraction of GBMs that develop from lower grade gliomas (LGGs) [2,30,31]. As a normal function, the IDH enzymes catalyze the oxidative carboxylation of isocitrate to α-ketoglutarate (α-KG) in combination with the reduction of nicotinamide adenine dinucleotide phosphate (NADP^+^) to NADPH. On the other hand, mutant IDH obtains a neomorphic activity that converts a-KG to d-2-hydroxyglutarate (d-2-HG) in an NADPH-consuming reduction, which competitively inhibits a-KG-dependent dioxygenases, eventually shifting the genome-wide histone and DNA methylome in gliomas [32,33]. These epigenetic changes are considered to lock tumor cells in an immature state [34], but the association of *IDH* mutations and invasive phenotypes remain to be clarified. Although GBM with a mutant *IDH* gene (GBM, *IDH*-mutant) have a better prognosis than high grade gliomas with wildtype *IDH* [35], the strong correlation between *IDH1* mutational status and the invasive characteristics was observed based on MRI (magnetic resonance imaging) studies [36]. Interestingly, the neurotransmitter glutamate in the brain may act as a chemotactic compound, specifically for *IDH*-mutated glioma cells [37]. In addition, overexpression of mutant *IDH2* (R172G) in glioma cells induces nuclear accumulation of β-catenin and upregulation of HIF-1α (hypoxia-inducible factor-1α) signaling that were closely related with tumor invasion and chemoresistance [38]. Alternatively, *IDH* mutations promote gliomagenesis by disrupting chromosomal topology and allowing aberrant regulatory interactions that induce oncogene expression such as PDGFR (platelet-derived growth factor receptor) [39], the combination of which is frequently observed in the proneural subtype of GBM [40,41]. Of note, PDGF signaling significantly promotes migratory capacity of glioma cells [42,43]. Tumor cells demonstrate highly invasive features in PDGF-induced glioma models, and PDGF-induced tumor cells transform surrounding NG2 proteoglycan-positive glial progenitors into migratory morphology in a paracrine fashion (Figure 2). Further, a number of Iba-1-positive microglia are observed in the invasive front of PDGF-induced gliomas, and microglial cells could also transform NG2-positive glial progenitors into migratory morphology (Figure 2). The action of PDGF signaling thus may alternatively explain the invasive phenotypes of *IDH*-mutant glioma cells. Future studies would be necessary to further clarify the association between *IDH* mutation, epigenetic changes (G-CIMP: glioma-CpG island methylator phenotype) [33], and glioma cell invasion.

### 3.2. Invasive Phenotypes in IDH-Wildtype Gliomas

Recent progresses in multi-disciplinary molecular analyses of cancers, based on large-scale DNA methylation profiling and next-generation sequencing approaches, have led to the molecular stratification of GBM by the combination of molecular genetic signatures. The Cancer Genome Atlas (TCGA) Research Network has generated the comprehensive catalog of genomic abnormalities driving tumorigenesis and has clarified three core pathways in GBM: (1) RTK/RAS/PI3K signaling, (2) p53 and (3) Rb pathways [44,45]. Among these, the genomic characterization of GBM with a wildtype *IDH* gene (GBM, *IDH*-wildtype) reveals frequent genetic alterations of the growth factor receptor-PI3K-Akt signaling pathway that activate mammalian target of rapamycin (mTOR) signaling [45]. Interestingly, RTK-PI3K/Akt-mTOR pathways are well known to promote invasive phenotypes of the glioma cells (Figure 2).

Epidermal growth factor receptor (*EGFR*, located on chromosome 7p12) amplification is a hallmark of GBM, specifically primary tumors [46,47]. About 50% of *EGFR*-amplified GBM express a ligand-independent truncated mutant variant, EGFRvIII, which is characterized by genomic deletion of exons 2–7, resulting in a constitutively active oncogenic form [47]. The presence of *EGFR* mutations significantly promotes the invasive capacity of glioma cells through the regulation of integrin [48,49], CAMs [50], urokinase-type plasminogen activator/receptor (uPA/uPAR) [51,52], MMPs [53,54] and microRNAs [55,56]. Wild-type EGFR is reported to be involved in the switch between invasive and angiogenic phenotypes in GBM [57]. EGFR pathway may also be associated with epithelial-mesenchymal transition (EMT) in gliomas [58], which plays a key role in cancer invasion and metastasis [59]. Located downstream of EGFR signaling, we have revealed the important role of mTOR complex 2 (mTORC2) in glioma pathogenesis through chemoresistance and metabolic reprogramming [60,61]. Of note, mTORC2 may be involved in the invasive phenotype of cancer cells [62,63,64] since mTORC2 functions upstream of Rho GTPases to organize the actin cytoskeleton [65,66]. Further studies are needed to elucidate the involvement of mTORC2 in the invasiveness of glioma cells with an activation of the EGFR-PI3K/Akt-mTOR pathway.

### 3.3 Metabolic Reprogramming as a Driving Force of Glioma Cell Invasion

**Figure 3:**
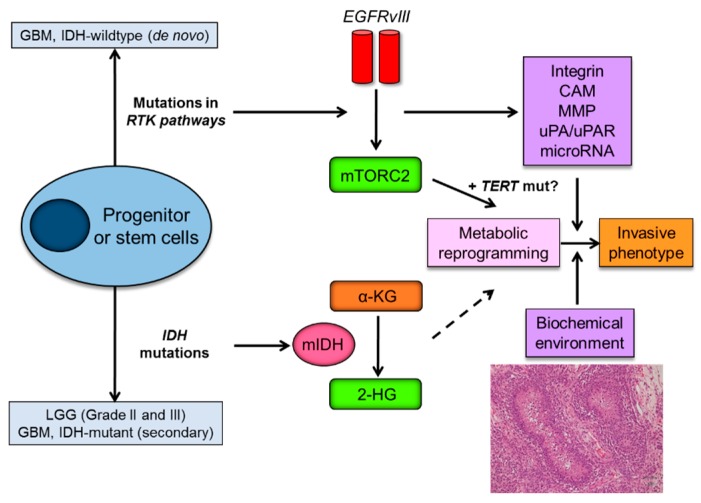
Metabolic reprogramming as a driving force of glioma invasive phenotype. Mutations in *IDH* that are identified as an early genetic event in grade II/III LGG and secondary GBM, and genetic alterations of key components of the growth factor receptor-PI3K-Akt-mTOR, which are main genetic aberrations in de novo GBM, play an essential role in metabolic reprogramming in diffuse gliomas. Metabolic reprogramming by mTORC2 (also possibly by TERT) could contribute to invasive phenotypes of glioma cells, in combination with pro-invasive molecules produced by EGFR signaling pathways. Biochemical microenvironment such as necrosis (hypoxia) also significantly affects the migratory capacity of the glioma cells. IDH, isocitrate dehydrogenase; LGG, lower grade glioma; GBM, glioblastoma; RTK, receptor tyrosine kinase; EGFRvIII, epidermal growth factor receptor variant III; mTORC2, mammalian target of rapamycin complex 2; mIDH, mutant form of IDH enzymes; α-KG, α-ketoglutarate; 2-HG, 2-hydroxyglutarate; TERT, telomerase reverse transcriptase; mut, mutation; CAM, cell adhesion molecule; MMP, matrix metalloproteinase; uPA, urokinase receptor.

Metabolic reprogramming or “the Warburg effect” is re-emerging as a central hallmark of cancer [67]. Cancer cells demonstrate a unique metabolism to convert the majority of glucose into lactate even in the presence of sufficient oxygen. Of interest, emerging evidences support the role of metabolic reprogramming in cancer invasive phenotypes. For instance, metabolic reprogramming supports the invasive phenotype in malignant melanoma [68], and metabolic stress regulates cytoskeletal dynamics and metastasis of cancer cells [69]. Importantly, major genetic and signaling aberrations in *IDH*-wildtype GBM including mTORC2 (in the EGFR-mTOR pathway) and TERT (telomerase reverse transcriptase) play an important role in transcriptional regulation and metabolic reprogramming [70,71], possibly linking genetic mutations and invasive phenotypes through metabolic reprogramming (Figure 3).

Metabolic reprogramming results in changes in intracellular nutrient levels, which can affect oncogenic signaling via control of epigenetics as well as post-translational modifications of cytoplasmic proteins [72,73,74]. The findings suggest the involvement of intermediary metabolites in the important cellular functions in cancer cells including cell migration. Glycolytic metabolites, such as glucose-6-phosphatase, are key metabolic regulators of GBM invasion [75]. NAD(H), a product in glycolysis and TCA cycle, regulates cell motility coupled with pyruvate-lactate conversion by lactate dehydrogenase (LDH) and with changes in intracellular and extracellular pH [76]. Of note, recent studies demonstrated the involvement of NAD^+^ metabolism in *IDH1* mutant cancers [77], suggesting the role of metabolic reprogramming in cancer cell invasion even in *IDH*-mutant gliomas. Glutaminolysis is an essential anaplerotic part of the cancer metabolism, and glutamate is produced by the tumor from glutamine and released through the cystine-glutamate antiporter xCT, which is recently reported to be regulated by mTORC2 [78]. Glioma cells utilize glutamate to promote invasion by inducing oscillatory intracellular Ca^2+^ changes through activation of Ca^2+^-permeable AMPA receptors [3,79].

From a morphological standpoint, “microvascular proliferation” and “palisading necrosis” are diagnostic hallmarks of GBM, which could also establish specific metabolic microenvironment/niche for glioma cells. In human GBM surgical specimens, tumor cells palisading around necrotic areas are exposed to hypoxic stimuli and induce dramatic upregulation of HIF-1α. Interestingly, GBM cells around necrosis often display migratory morphology, which may implicate hypoxia-regulated migration toward or away from the necrotic regions [80,81], and limitations in oxygen diffusion would be further affected by abnormal tumor vasculatures. Experimental models support the idea that tumor hypoxia results in increased GBM cell migration, and GBM cells respond to hypoxia with an induction of c-Met, which is the receptor for hepatocyte growth factor to secrete uPA and MMPs for avidly degrading ECM and invading the surrounding tissue [81].

## 4. Conclusions and Outlook

The traditional histological classification of diffuse gliomas has been replaced by the genetics-oriented classification system based on recent identification of distinct genetic and epigenetic features. Through the efforts to clarify the link between genetic aberrations and glioma biology, “the genotype-phenotype correlation” will be unveiled for further refinement of glioma classification. One of the key mechanisms to link the genetic aberrations with glioma pathogenesis is through cancer metabolic reprogramming. Genetic aberrations render glioma cells to increase glucose uptake to meet the increased energetic and biosynthetic demands imposed by rapid tumor growth, modulate epigenetic landscapes and eventually drive tumor progression [82]. Interestingly, cell proliferation and migration seem to be a dichotomic phenomenon, and glioma cells may utilize metabolic reprogramming for proliferation in the center of the tumor tissue and for infiltration in the periphery, both of which are a key characteristic of diffuse glioma.

The propensity of glioma cells to move and invade the brain tissue is considered to be a malignant feature, but these biological traits may be inherited from their neural ancestors or residing cells. Therefore, caution should be made in treatment when trying to exploit the invasive nature of glioma cells. Therapeutic targets should not be the migratory system itself shared by cancer and inherent cells, but the upstream genetic, epigenetic and metabolic aberrations to regulate invasive phenotypes specific to cancer cells. Additionally, therapeutic strategies should take into consideration not only the intracellular reprogramming of metabolism in cancer cells, but the biochemical environment that can affect the infiltrative behavior of tumor cells in a genotype-specific fashion, potentially by shifting the relative fitness of cells bearing a mutation to grow and invade within that metabolic niche. Future studies are needed to determine precisely how chief genetic mutations and subsequent epigenetic changes in diffuse glioma facilitate invasive phenotype through cancer metabolic reprogramming in combination with extracellular environment, in order to orchestrate these insights into more effective treatments for glioma patients.
